# A Handbook on Leprosy

**Published:** 1896-12

**Authors:** 


**A Handbook on Leprosy.** By S. P. Impey, M.D. Pp. xv., 116. London: J. & A. Churchill. 1896.

We have examined this work with great interest, because it is not long since we reviewed a work by Drs. Hansen and Looft, relating to leprosy as seen in Norway and Sweden. Dr. Impey, who was lately the Medical Superintendent of the Robben Island Leper and Lunatic Asylums, Cape Colony, deals with the incidence of the disease in South African States, where the conditions of climate are so very different, yet we note that the general results of leprosy are very similar in both these parts of the world. This condition of things corroborates a statement made by Dr. Impey (p. 27), that "no external influences, climatic, telluric, or meteorological, have any appreciable effect on the spread of leprosy."

The authors of both works agree that, practically, leprosy exists in two forms—the tubercular and the anæsthetic, and that there are mixed cases. Dr. Impey, in addition, describes a form, "syphilitic leprosy": five very characteristic plates are given in illustration, and we find that two such dreadfully disfiguring diseases do not neutralise each other; nay, rather they intensify each other's attack upon the system by combining in a terrible partnership. Altogether Dr. Impey gives us thirty-seven splendidly-executed plates, illustrating the disease in its various stages and forms, and adds a map of Cape Colony, showing the geographical distribution of the disease.

This work is a valuable contribution to science. It shows that leprosy knows no distinction of sex, although more cases occur amongst males; but they are more exposed to predisposing conditions. Then we have strong evidence that it is not infectious, and that hereditariness counts for very little. Thus in Robben Island there were 266 leper-parents, who had had 951 children, of whom only 23, or about 2.43 per cent., became leprous. Of 520 lepers in the Asylum, 475 were born of healthy parents; and of the remaining 45 cases, in 25 the father was alone affected, in 16 the mother, and both parents in 4 only. Leprosy may attack all ages—from 3 to 80 years—the average being about 29. About 2,000 cases were estimated to exist in the South African States. Chapters XIX. and XX. deal with isolation, according to the present system, and suggest some desirable modifications. The regulations framed by Government for private isolation are given in the last chapter.

